# Addressing overdose risk among unstably housed women in San Francisco, California: an examination of potential fentanyl contamination of multiple substances

**DOI:** 10.1186/s12954-020-00361-8

**Published:** 2020-03-10

**Authors:** Meredith C. Meacham, Kara L. Lynch, Phillip O. Coffin, Amanda Wade, Eliza Wheeler, Elise D. Riley

**Affiliations:** 1 Department of Psychiatry and Weill Institute for Neurosciences, University of California San Francisco, San Francisco, USA; 2 Department of Laboratory Medicine, University of California San Francisco, San Francisco, USA; 3 San Francisco Department of Public Health, San Francisco, USA; 4 Department of Medicine, Division of HIV, Infectious Diseases and Global Medicine, University of California San Francisco, San Francisco, USA; 5 Harm Reduction Coalition, Oakland, USA

**Keywords:** Fentanyl, Crack cocaine, Methamphetamine, Homelessness, Overdose, Women

## Abstract

**Background:**

Numerous reports have led to concerns that fentanyl is added to many street drugs as an adulterant, including to stimulants like cocaine and methamphetamine, and could increase risks for negative health outcomes.

**Methods:**

We collected information regarding recent substance use through self-report and urine toxicology (confirmed with mass spectrometry) once a month for up to 6 monthly study visits from a probability sample of 245 women in San Francisco with a history of housing instability (2016-2019). We compared the presence of fentanyl metabolites with (1) the presence of metabolites for other substances and (2) self-reported past week substance use.

**Results:**

Out of 1050 study visits, fentanyl metabolites were detected 35 times (i.e., at 3% of all study visits and among 19/245, or 8% of all women). In most but not all (91%, or 32/35) of these detected cases, heroin or opioid medication use was self-reported. Among women who reported cocaine or methamphetamine use, but did not use heroin or opioid medication, fentanyl was detected in only 1 of 349 cases (0.3%). In adjusted logistic regression, the presence of fentanyl metabolites was independently associated with (1) presence of opiate, heroin, and benzodiazepine metabolites, and (2) self-reported past week use of heroin and opioid medications. Fentanyl metabolite detection was not independently associated with cocaine or methamphetamine use.

**Conclusions:**

The presence of fentanyl metabolites in this population was almost entirely among women who also reported using heroin or opioid pills. These data do not support the hypothesis that fentanyl is being routinely added to stimulants as an adulterant on a large scale in this region.

## Background

In the USA, nearly a third (31%) of the estimated 63,632 overdose deaths in 2016 involved synthetic opioids like illicit fentanyl [1], which is 30-50 times more potent than heroin. In 2017, there were an estimated 70,237 overdose deaths and age-adjusted rates of overdose deaths involving any synthetic opioids rose by 45.2% from 2016 [2]. Some reports have led to concerns regarding the large-scale contamination of street drugs, including stimulants like cocaine and methamphetamine, with fentanyl and fentanyl analogs [3–8]. If true, this could increase the risk of overdose among stimulant-using populations who may be opioid-naive or have lower tolerance for opioids, and who may not receive targeted interventions or take the same precautions as primarily heroin or opioid-using populations. However, the extent of possible fentanyl contamination is relatively unknown due to a dearth of systematic evidence.

Estimating prevalence of fentanyl exposure and its association with other drugs in community settings would provide a better understanding of population-level health risks. This is a crucial step toward expanding health care guidelines and policies that address the recent increases in fentanyl-related overdose [9]. Several recent studies, based in New York City, Massachusetts, British Columbia, and Canada, found higher rates of fentanyl detected from hair and urine samples compared to self-reported fentanyl use [10–14]. These studies suggest that fentanyl is being used to adulterate other drugs; however, they do not focus specifically on women or people who use stimulants. Impoverished women who use drugs face vulnerability to drug-related harms differently than do men, due to factors such as gendered violence and gendered norms regarding drug use, sex, and reproductive health [15–17].

San Francisco and California as a whole have not been as impacted by fentanyl as the Eastern United States [2], yet rates of opioid overdose deaths have been increasing statewide. In San Francisco, there was a doubling of fentanyl-related overdose deaths, from 11 individuals in 2015 to 22 in 2016. Several clusters of overdoses potentially related to fentanyl occurred in 2015 (the year fentanyl is believed to have entered the San Francisco market), 2017, and 2018 [5–8].

Recent spikes in fentanyl-related deaths and detection of fentanyl in drug samples [4–6] have raised concerns among health officials and harm reduction service providers, who have worked to create proactive public health messaging and community-engaged responses. The purpose of this study was to address these concerns by examining the presence of metabolites for multiple substances, including fentanyl, from a probability sample of community-recruited adult women with a history of housing instability living in San Francisco. We then compared the presence of fentanyl metabolites with [1] the presence of metabolites for other substances and with [2] self-reported past week substance use.

## Methods

### Study population

The data for this analysis come from the PULSE (Polysubstance Use and Health Outcomes Evaluation) study, a longitudinal bio-behavioral study examining the impact of polysubstance use and HIV on the cardiac health of women. Eligibility criteria included female sex at birth, being at least 18 years old, and having a history of housing instability (slept on the street, in a homeless shelter, or with a series of acquaintances [“couch surfed”] because there was no other place to sleep). Women living with HIV were oversampled to meet HIV-specific study aims. Participants were recruited from a probability sample of street encampments, homeless shelters, free meal programs, single room occupancy (SRO) hotels, and a public HIV clinic in San Francisco, California. Longitudinal information from 245 study participants was collected in six monthly study visits between 2016 and 2019. Participants were reimbursed $40 for each interview completed. Study procedures were approved by the Institutional Review Board of the University of California, San Francisco.

### Measures

At each study visit, participants completed a questionnaire with a trained interviewer and provided a urine sample. The main outcome of interest was detection of fentanyl metabolites (fentanyl or norfentanyl) via urine toxicology. Urine toxicology also assessed presence of metabolites for opiates, oxycodone, tramadol, heroin, cocaine, methamphetamine, and benzodiazepines. Metabolite presence was confirmed with liquid chromatography tandem mass-spectrometry (LC-MS/MS).

Self-reported use of opioids, heroin, crack cocaine, powder cocaine, and benzodiazepines was assessed at each visit via audio computer-assisted self-interview (ACASI). If participants reported ever using a substance, they were asked the time of last use, with response options including the last week, month, year, and over a year ago. Opioids were described as “opiate pain killers like Codeine, fentanyl, Vicodin, OxyContin, Percocet or morphine.” Past week substance use was compared with fentanyl detection, as this was the shortest period of time that would be most comparable with the fentanyl detection period of approximately 3 days.

Socio-demographic characteristics assessed at baseline included age, race, Latina ethnicity (yes/no), education (completion of high school – yes/no), and homelessness (yes/no).

### Data analysis

Data were analyzed at the study visit level (i.e., out of all assessments) rather than individual level (i.e., out of 245 women) because participants may have procured substances from different places each month in the context of a highly variable and unpredictable drug supply. Unadjusted associations between the presence of fentanyl metabolites and metabolites for other substances or self-reported past week substance use were estimated with logistic regression. Multivariable logistic regression models were used to determine independent associations in two separate models (i.e., one for metabolites and one for self-reported use). Analyses were conducted in R version 3.4.3.

## Results

Among 245 unstably housed women, baseline self-reported past week use of stimulants (i.e., crack or powder cocaine, methamphetamine) (53.1%) was more common than use of heroin or opioid medications (33.1%) (Table 1). The average age was 51.6 years (SD, 10.8) and the majority were women of color (Black/African American, 40.8%; White/Caucasian, 26.5%; Multiracial, 19.2%; Native American/Alaskan Native, 6.5%; Other, 3.7%; Asian/Pacific Islander, 3.3%; Latina ethnicity, 15.1%). About 7 in 10 women had completed high school (69.8%) and while all had a history of homelessness or housing instability, 38.8% were experiencing homelessness at the time of the baseline survey. Almost one-third of participants were living with HIV (31.4%).

**Table 1:** Sample description at baseline assessment (*N* = 245 adult women with a history of unstable housing)

	*N*	Percent
*Social determinants and HIV status*		
Age (mean, SD)	51.6	10.8
Race		
Native American/Alaskan Native	16	6.5
Asian/Pacific Islander	8	3.3
Black/African American	100	40.8
White/Caucasian	65	26.5
Other	9	3.7
Multiracial	47	19.2
Latina	37	15.1
Completed high school	171	69.8
Experiencing homelessness	95	38.8
HIV+	77	31.4
*Past week substance use*		
Opioids (painkiller medications, heroin)	81	33.1
Stimulants (crack, cocaine, methamphetamine)	130	53.1

Out of 1050 study visits, fentanyl metabolites were detected 35 times (among 8% of all women and 3% of all study visits). In most but not all (91%, or 32/35) cases of fentanyl detection, heroin or opioid medication use was also self-reported. Among women who reported past week use of cocaine or methamphetamine, but did not report past week use of heroin or opioid medication, fentanyl was detected in only 1 of 349 cases (0.3%). In unadjusted logistic regression, the odds of fentanyl detection were significantly greater when opiates, heroin, methamphetamine, or benzodiazepines were detected. In adjusted analysis with all substance metabolites, only metabolites from opiates (AOR = 4.90, 95% CI, 2.23-11.01), heroin (AOR = 5.41, 95% CI, 1.85-15.12), and benzodiazepines (AOR = 2.72, 95% CI, 1.12-613) were associated with fentanyl metabolite detection (Table 2).

**Table 2:** Associations between the presence of fentanyl metabolites and (a) other substance metabolites detected by urine toxicology and (b) self-reported past week substance use (*N* = 1050 study assessments)

	Detection period	Study visits detected *N* (%)	Co-occurring fentanyl detection	Unadjusted odds of fentanyl detection	95% confidence interval	Adjusted odds of fentanyl detection	95% confidence interval
(*a*) *Metabolites detected by urine toxicology*							
Fentanyl	3 days	35 (3%)	--	--	--	--	--
Opiates (codeine, etc.)^1^	2-3 days	221 (21%)	23 (10%)	**7.91**	**1.37-16.67**	**4.90**	**2.23-11.01**
Oxycodone/Oxymorphone^2^	1-3 days	51 (5%)	4 (8%)	2.65	0.77-7.05	1.53	0.41-4.59
Tramadol^3^	1-2 days	110 (11%)	5 (5%)	1.44	0.48-3.49	1.57	0.50-4.13
Heroin	12-24 hours	28 (3%)	8 (29%)	**14.74**	**5.68-35.47**	**5.41**	**1.85-15.12**
Cocaine (crack or powder)	2-3 days	709 (68%)	24 (3%)	1.71	0.85-3.67	1.36	0.64-3.07
Methamphetamine	2-4 days	293 (28%)	17 (6%)	**2.53**	**1.27-4.99**	1.32	0.60-2.83
Benzodiazepines	Up to 2 weeks	112 (11%)	9 (8%)	**3.06**	**1.32-6.48**	**2.72**	**1.12-6.13**
(*b*) *Self-reported past week substance use*							
Opioids^4^	--	285 (27%)	29 (10%)	**14.30**	**6.29-38.54**	**9.26**	**3.83-25.95**
Heroin	--	103 (10%)	20 (19%)	**15.03**	**7.41-30.79**	**6.43**	**2.81-15.04**
Crack cocaine	--	422 (40%)	19 (5%)	1.80	0.92-3.60	1.38	0.61-3.09
Powder cocaine	--	109 (10%)	7 (6%)	2.23	0.88-4.98	0.71	0.24-1.90
Methamphetamine	--	188 (18%)	16 (9%)	**4.18**	**2.08-8.29**	1.75	0.76-3.93
Benzodiazepines^5^	--	171 (16%)	12 (7%)	**2.80**	**1.33-5.65**	1.02	0.24-1.90

^1^Includes morphine, codeine, hydrocodone, hydromorphone, dihydrocodeine, morphine glucuronide, codeine glucuronide

^2^Includes oxycodone, oxymorphone

^3^Includes tramadol, meperidine, normeperidine, levorphanol, O-desmethyl-cis-tramadol

^4^Could include codeine, fentanyl, Vicodin, OxyContin, Percocet or morphine

^5^Could include Valium, Librium, Xanax, or Ambien or other anti-anxiety drug

When compared to self-reported past week use of these substances, use of opioids, heroin, methamphetamine, and benzodiazepines was again significantly associated with fentanyl metabolite detection. When adjusting for all substances, only opioids (AOR = 9.26, 95% CI, 3.83-25.95) and heroin (AOR = 6.43, 95% CI, 2.81-15.04) use were significantly associated with fentanyl metabolite detection (Table 2).

## Discussion

The presence of fentanyl metabolites in this population was almost entirely among women who reported using heroin or opioid pain medications and was not associated with stimulant use in adjusted analysis. These data therefore do not support the hypothesis that fentanyl is being routinely added to stimulants as an adulterant in this region.

The different findings in self-reported vs. metabolite data for benzodiazepines in adjusted analyses may be explained by the longer detection period for benzodiazepines, although illicitly produced benzodiazepines may also be contaminated with fentanyl. Methamphetamine metabolite presence and self-reported use were associated with fentanyl presence in unadjusted analyses, but not in analyses adjusted for other substances. Given the potential confounding effects of multiple concurrent substance use, these findings emphasize the importance of adjusted analyses in future surveillance.

Two studies, based in British Columbia and Canada, have found evidence suggesting occasional fentanyl contamination of methamphetamine. Amlani et al. found that reported use of both fentanyl and methamphetamine was independently associated with fentanyl detection among harm reduction services participants, even after adjusting for concurrent opioid use [13]. In a drug-checking program in British Columbia, 90.6% of samples expected to be heroin and 5.9% of samples expected to be methamphetamine tested positive for fentanyl [14]. Exactly where in the supply chain [18–20] that fentanyl contamination of stimulants is occurring is unclear, but at the end of the supply chain is one possibility that is consistent with recent surveillance in Ohio. Examination of weight patterns in stimulant seizures from Ohio found that the rare instances of fentanyl contamination appear to occur in cocaine seizures of under 1 gram, and less often in methamphetamine seizures, suggesting that contamination of cocaine occurs at the end of the supply chain in that region [21].

The finding that only 0.3% of cases where women who did *not* report opioid use tested positive for fentanyl suggests that intentional contamination of stimulants with fentanyl is likely rare. Nevertheless, accidental exposure to fentanyl consumption with resultant overdose has been reported in San Francisco [6, 7]. The strong associations between opioid pain medications or heroin use and fentanyl detection echo prior research with opioid-using populations that reinforces the importance of community-based naloxone distribution programs [22, 23], fentanyl test strips [24, 25], drug-checking services [26], and supervised consumption or overdose prevention sites [27, 28], where overdose prevention services can be accessed. Although many people who use drugs are now aware of the possibility of fentanyl contamination, the regular application of overdose prevention measures is often inconsistent due to structural factors such as poverty and homelessness [29].

Study limitations include sampling from a single city, which limits generalizability to other locations, and a longer self-reported use period than drug detection periods. There was also no direct assessment of known fentanyl use as distinct from other opioid pain medication [30] for this secondary data analysis. Study strengths include longitudinal comparisons of both self-reported and mass-spectrometry toxicology-confirmed substance use, a focus on stimulant use, and a focus on women.

The current study found insufficient evidence for the contamination of stimulants with fentanyl, yet a strong relationship between fentanyl exposure and heroin and opioid pill use.

## Data Availability

The datasets during and/or analyzed during the current study available from the corresponding author on reasonable request.
